# Supplementary material for: Multicomponent Training and Optimal Dosing Strategies for Adults with Hypertension: A Systematic Review and Meta-Analysis of Randomized Controlled Trials
Source: Sports (Basel). 2023 Jun 8;11(6):115. doi: 10.3390/sports11060115 (PMC10304212; doi:10.3390/sports11060115)
Supplement: Supplementary file 1 [file sports-11-00115-s001.zip › sports-2421119-supplementary.pdf]

Supplementary material

Supplementary Table S1. Search strategy

PubMed	(((hypertension[Title]) OR (blood pressure[Title]) OR (systolic[Title]) OR (diastolic[Title]) OR (hypertensive[title])) AND ((aerobic*[Title]) OR (endurance*[Title]) OR (weight*[Title]) OR (HIIT[Title]) OR (MICT[Title]) OR (fitness([Title]) OR (resistance[Title]) OR (combined[Title]) OR (multicomponent[Title])))
Cochrane	(hypertension):ti AND (aerobic*):ti"<br>(hypertension):ti AND (endurance*):ti"<br>(hypertension):ti AND weight*):ti"<br>(hypertension):ti AND HIIT):ti"<br>(hypertension):ti AND MICT):ti"<br>(hypertension):ti AND fitness):ti"<br>(hypertension):ti AND resistance):ti"<br>(hypertension):ti Combined):ti"<br>(hypertension):ti multicomponent):ti"<br>(blood pressure):ti AND (aerobic*):ti"<br>(blood pressure):ti AND (endurance*):ti"<br>(blood pressure):ti AND weight*):ti"<br>(blood pressure):ti AND HIIT):ti"<br>(blood pressure):ti AND MICT):ti"<br>(blood pressure):ti AND fitness):ti"<br>(blood pressure):ti AND resistance):ti"<br>(blood pressure):ti Combined):ti"<br>(blood pressure):ti multicomponent):ti"<br>(systolic):ti AND (aerobic*):ti"<br>(systolic):ti AND (endurance*):ti"<br>(systolic):ti AND weight*):ti"<br>(systolic):ti AND HIIT):ti"<br>(systolic):ti AND MICT):ti"<br>(systolic):ti AND fitness):ti"<br>(systolic):ti AND resistance):ti"<br>(systolic):ti Combined):ti"<br>(systolic):ti multicomponent):ti"<br>(diastolic):ti AND (aerobic*):ti"<br>(diastolic):ti AND (endurance*):ti"<br>(diastolic):ti AND weight*):ti"<br>(diastolic):ti AND HIIT):ti"<br>(diastolic):ti AND MICT):ti"<br>(diastolic):ti AND fitness):ti"<br>(diastolic):ti AND resistance):ti"<br>(diastolic):ti Combined):ti"<br>(diastolic):ti multicomponent):ti"<br>(hypertensive):ti AND (aerobic*):ti"<br>(hypertensive):ti AND (endurance*):ti"<br>(hypertensive):ti AND weight*):ti"<br>(hypertensive):ti AND HIIT):ti"<br>(hypertensive):ti AND MICT):ti"<br>(hypertensive):ti AND fitness):ti"<br>(hypertensive):ti AND resistance):ti"<br>(hypertensive):ti Combined):ti"<br>(hypertensive):ti multicomponent):ti"

WOS	(TI=(hypertension)) AND TI= (aerobic*)<br>(TI=(hypertension)) AND TI= (endurance*)<br>(TI=(hypertension)) AND TI=(weight*)<br>(TI=(hypertension)) AND TI=(HIIT)<br>(TI=(hypertension)) AND TI=(MICT)<br>(TI=(hypertension)) AND TI=(fitness)<br>(TI=(hypertension)) AND TI=(resistance)<br>(TI=(hypertension)) AND TI=(Combined)<br>(TI=(hypertension)) AND TI=(multicomponent)<br>(TI=(blood pressure)) AND TI=(aerobic*)<br>(TI=(blood pressure)) AND TI= (endurance*)<br>(TI=(blood pressure)) AND TI=(weight*)<br>(TI=(blood pressure)) AND TI=(HIIT)<br>(TI=(blood pressure)) AND TI=(MICT)<br>(TI=(blood pressure)) AND TI=(fitness)<br>(TI=(blood pressure)) AND TI=(resistance)<br>(TI=(blood pressure)) AND TI=(Combined)<br>(TI=(blood pressure)) AND TI=(multicomponent)<br>(TI=(systolic)) AND TI=(aerobic*)<br>(TI=(systolic)) AND TI= (endurance*)<br>(TI=(systolic)) AND TI=(weight*)<br>(TI=(systolic)) AND TI=(HIIT)<br>(TI=(systolic)) AND TI=(MICT)<br>(TI=(systolic)) AND TI=(fitness)<br>(TI=(systolic)) AND TI=(resistance)<br>(TI=(systolic)) AND TI=(Combined)<br>(TI=(systolic)) AND TI=(multicomponent)<br>(TI=(diastolic)) AND TI= (aerobic*)<br>(TI=(diastolic)) AND TI= (endurance*)<br>(TI=(diastolic)) AND TI=(weight*)<br>(TI=(diastolic)) AND TI=(HIIT)<br>(TI=(diastolic)) AND TI=(MICT)<br>(TI=(diastolic)) AND TI=(fitness)<br>(TI=(diastolic)) AND TI=(resistance)<br>(TI=(diastolic)) AND TI=(Combined)<br>(TI=(diastolic)) AND TI=(multicomponent)<br>(TI=(hypertensive)) AND TI=(aerobic*)<br>(TI=(hypertensive)) AND TI=(endurance*)<br>(TI=(hypertensive)) AND TI=(weight*)<br>(TI=(hypertensive)) AND TI=(HIIT)<br>(TI=(hypertensive)) AND TI=(MICT)<br>(TI=(hypertensive)) AND TI=(fitness)<br>(TI=(hypertensive)) AND TI=(resistance)<br>(TI=(hypertensive)) AND TI=(Combined)<br>(TI=(hypertensive)) AND TI=(multicomponent)
EBSCO	TI hypertension AND TI aerobic*<br>TI hypertension AND TI endurance*<br>TI hypertension AND TI weight*<br>TI hypertension AND TI HIIT<br>TI hypertension AND TI MICT<br>TI hypertension AND TI fitness<br>TI hypertension AND TI resistance<br>TI hypertension AND TI Combined<br>TI hypertension AND TI multicomponent<br>TI blood pressure AND TI aerobic*

	TI blood pressure AND TI endurance*<br>TI blood pressure AND TI weight*<br>TI blood pressure AND TI HIIT<br>TI blood pressure AND TI MICT<br>TI blood pressure AND TI fitness<br>TI blood pressure AND TI resistance<br>TI blood pressure AND TI Combined<br>TI blood pressure AND TI multicomponent<br>TI systolic AND TI aerobic*<br>TI systolic AND TI endurance*<br>TI systolic AND TI weight*<br>TI systolic AND TI HIIT<br>TI systolic AND TI MICT<br>TI systolic AND TI fitness<br>TI systolic AND TI resistance<br>TI systolic AND TI Combined<br>TI systolic AND TI multicomponent<br>TI diastolic AND TI aerobic*<br>TI diastolic AND TI endurance*<br>TI diastolic AND TI weight*<br>TI diastolic AND TI HIIT<br>TI diastolic AND TI MICT<br>TI diastolic AND TI fitness<br>TI diastolic AND TI resistance<br>TI diastolic AND TI Combined<br>TI diastolic AND TI multicomponent<br>TI hypertensive AND TI aerobic*<br>TI hypertensive AND TI endurance*<br>TI hypertensive AND TI weight*<br>TI hypertensive AND TI HIIT<br>TI hypertensive AND TI MICT<br>TI hypertensive AND TI fitness<br>TI hypertensive AND TI resistance<br>TI hypertensive AND TI Combined<br>TI hypertensive AND TI multicomponent
